# Visible induced luminescence reveals invisible rays shining from Christ in the early Christian wall painting of the Transfiguration in Shivta

**DOI:** 10.1371/journal.pone.0185149

**Published:** 2017-09-26

**Authors:** Ravit Linn, Yotam Tepper, Guy Bar-Oz

**Affiliations:** 1 Conservation of Material Culture Heritage Unit, Department of Archaeology, University of Haifa, Mount Carmel, Haifa, Israel; 2 Zinman Institute of Archaeology, University of Haifa, Mount Carmel, Haifa, Israel; Oregon State University, UNITED STATES

## Abstract

The Transfiguration scene depicted in a Byzantine church at Shivta, Israel, is one of two figurative examples of the scene from the early Christian period. The use of Egyptian blue pigment in the wall painting was investigated with various analytical methods. Visible Induced Luminescence (VIL) imaging was used *in-situ* in order to map the distribution of the Egyptian blue pigment in the painting. The VIL imaging revealed surprising insights into the understanding of the iconography and the technology of this rare painting. Previously undetected elements of the painting include rays of light that were discovered emerging from the body of Christ and illuminating the other figures in the painting. Although this motif is an important part of the Transfiguration narrative and appears in most of its scenes depicted elsewhere, it had not been previously identified in this painting as it was undetectable by any other inspection technique. Another important result is the identification of Egyptian blue as a common blue pigment used at Shivta during the Byzantine period, when it is considered to be very rare.

## Introduction

The Transfiguration is the moment when Christ revealed his divine nature to his disciples, Peter, John and James [1]. The scene is described in all three synoptic Gospels (Matt. 17:1–13, Mark 9:2–13, and Luke 9:28–30), and has played a major role in debates about the nature of Christ since the first centuries of Christianity [2,3,4,5].

**Fig 1:**
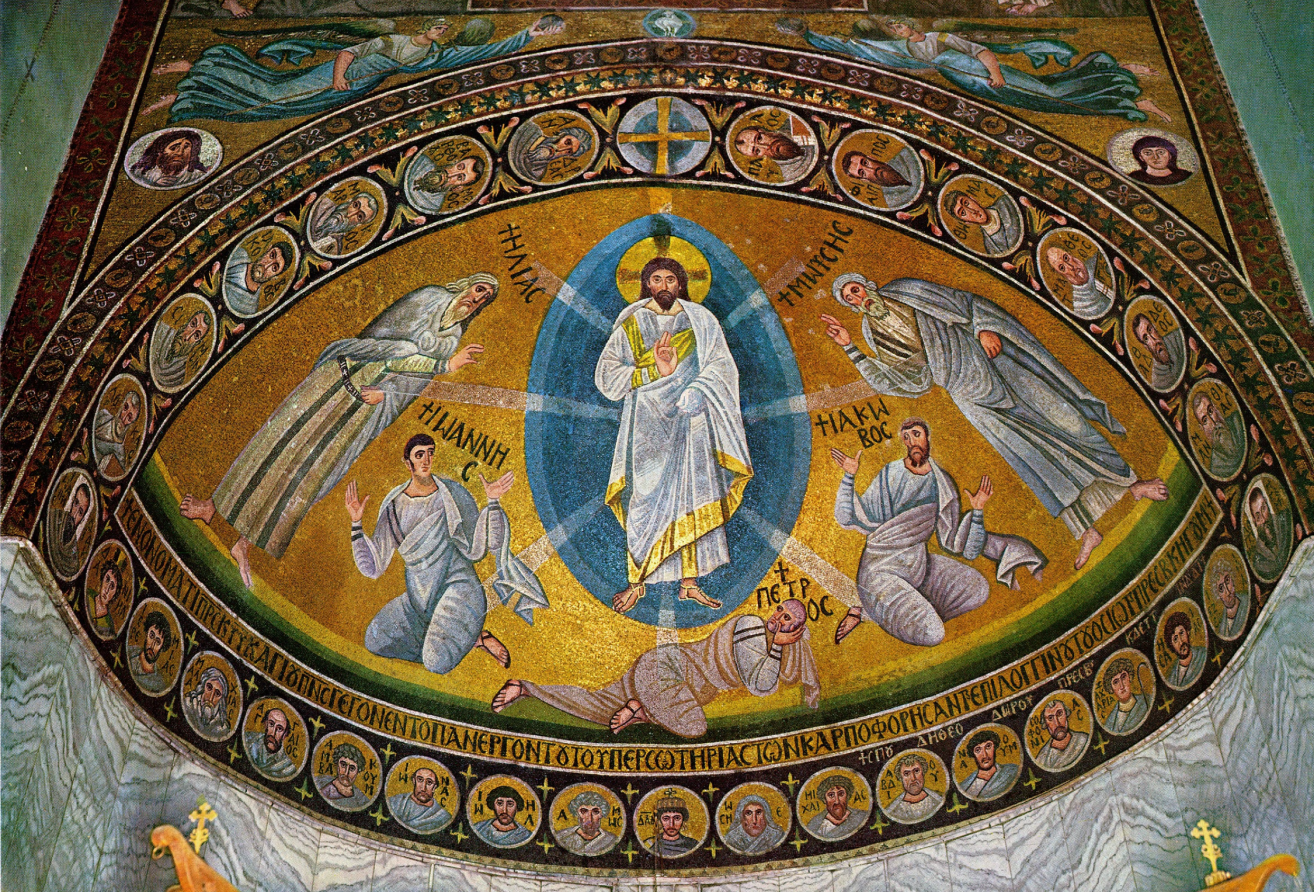
The apse mosaic of the Transfiguration scene from St. Catherine monastery in Sinai. This very well preserved composition of the Transfiguration, shows all its motives. Note the rays of light that emerge from Christ to the other figures. Reprinted from: George H. Forsyth and Kurt Weitzmann, The Monastery of Saint Catherine at Mount Sinai: The Church and Fortress of Justinian; plate CIII. Under a CC BY license, with permission from: The University of Michigan Press, Ann Arbor, 1973.

Very few *in-situ* Transfiguration images have survived from the period before Iconoclasm [1]. The best known example, an apse mosaic dated to 548–565 CE, is in the monastery of St. Catherine on Mount Sinai [1,6] (Fig 1).

Another example of a mosaic of the Transfiguration is in St. Apollinaire at Ravenna, in which Christ and the disciples are not presented as human figures. The Transfiguration scene in Shivta, based on its style and iconography is dated to the early 6th century CE. Besides the St. Catherine mosaic, it is the only other figurative scene of that era and the oldest known example executed as a wall painting [1,7].

**Fig 2:**
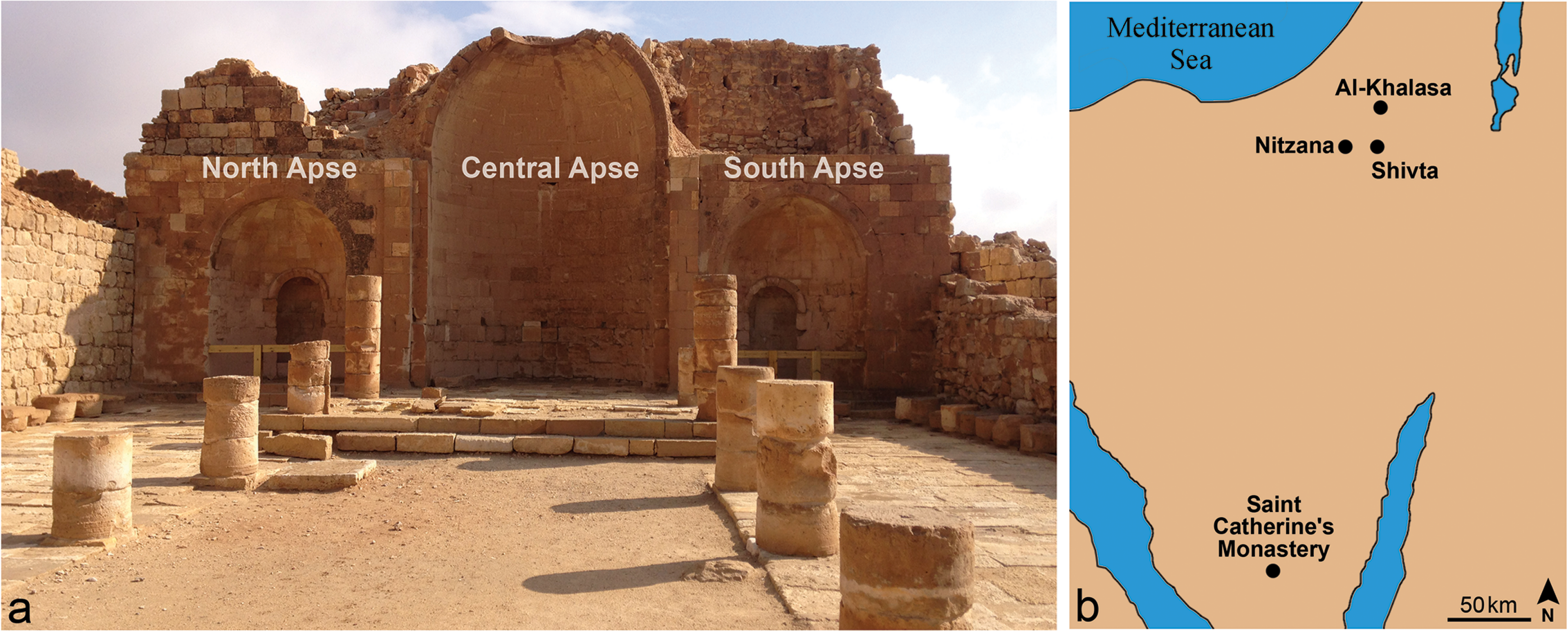
Location map of Shivta and view of the apses of the southern church. General view of the three apses of the southern church at Shivta (2a) (Photo: R. Linn, 2016). Map of the area of Shivta (2b).

Shivta, a UNESCO world heritage site, is located in the Negev desert in southern Israel, 43 km south-west of the city of Beer-Sheva (Fig 2). In the 4th century CE, Shivta became a large Byzantine village. It reached its peak during the 6th and 7th centuries and declined dramatically in the 8th and 9th centuries [8,9,10,11].

Three Byzantine churches have been found at Shivta along the north–south axis of the site, all named by their location: the northern church, the central church and the southern church (Fig 2A). All the churches were most probably decorated with wall paintings, as is evident from the limited remains that are still observed *in-situ* [12,13,14].

**Fig 3:**
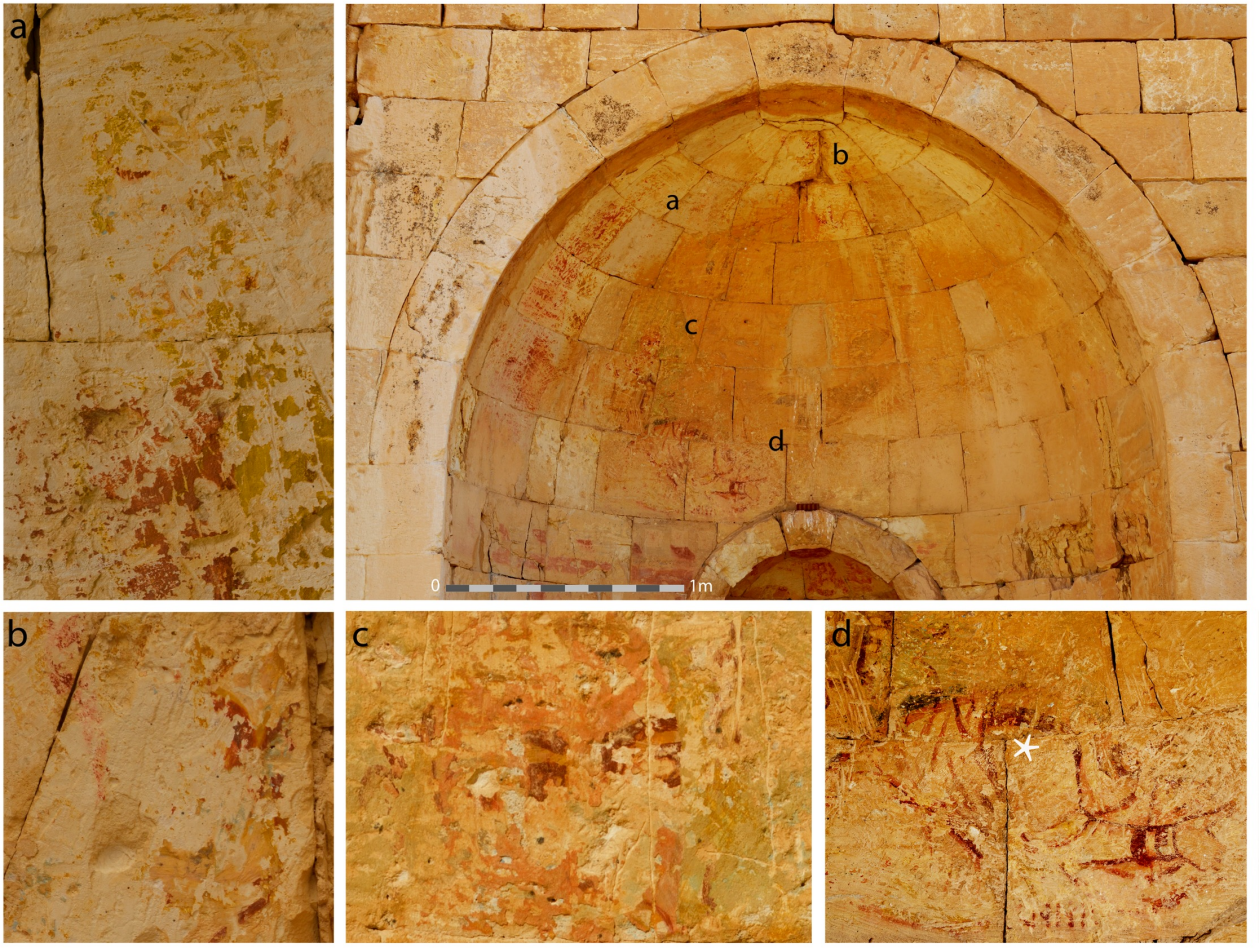
The southern apse of the southern church at Shivta. The painting survived in the upper part of the apse. Note its fragmentary condition and its hardly visible colors and iconography (Photo: R. Linn, 2016). Details of the painting include: 3a: The figure on the left of Christ. 3b: The face of Christ. 3c: The upper head of Peter. 3d: The entire figure of John (note the white star that indicates where sample no. Sh-3 was taken from).

The Transfiguration scene in the southern church is the only reported example that can still reveal parts of its original iconography, despite its extreme fragmentary condition (Fig 3). The scene was painted on the upper hemisphere of the southern apse, 2.5 m. in width and 2.0 m. in height. Most of the colors and details of the painting are preserved in the northern half of the apse, while in the southern half there are only scarce traces of paint (Fig 3).

The figures are painted in flesh tones, from dark pink to light pink, beige and white. The contour lines of the figures and the details of the faces and hands are in dark red, brown and yellow. The colors in the background surrounding the figures are green, beige, light yellow, pink and red. The blue color appears in many areas of the painting, mainly in the lower layer, and is therefore not very conspicuous (Fig 3).

This painting has been reported by very few scholars in the past, who mainly described its remaining iconography, including Woolley and Lawrence in 1914 (see S1 Text), [14], and more recently Figueras in 2006 [7] and Leatherbury in 2016 [1].

A detailed description of the remaining figures was given by Figueras [7] and Leatherbury [1]: John is a prostrate figure in the lowest left part of the painting (Fig 3D), identified by his name in Greek letters. Peter appears to be kneeling behind John (Fig 3C). Christ is depicted with a halo in the center of the painting (Fig 3B), inside a mandorla of light, with Moses and Elijah at either side (Fig 3A).

Although the painting in Shivta is extremely fragmentary and many details are completely missing, it still preserves some significant remains which provide important evidence of its motifs, design and composition. An initial examination of the painting, including instrumental analyses, demonstrated the use of Egyptian blue pigment and therefore the Visible Induced Luminescence (VIL) technique was selected as a major investigation method for this research.

VIL technique is based on the fact that Egyptian blue pigment luminesces strongly when excited by visible light [15,16]. The technique has been proposed as a method to detect and map Egyptian blue on works of art and archaeological finds [16,17,18]. It has been used on painted sculptures, museum artifacts and wall paintings, mainly in museums and laboratories [15,19,20,21].

Egyptian blue is the oldest synthetic pigment known, consisting of calcium copper silicate (CaCuSi_4_O_10_). It was commonly used throughout most of antiquity as a pigment in paintings, tombs, mummies, coffins, ceramic glazing and wall paintings [22,23,24,25]. From the Byzantine period, (the 4th century CE onward), its use decreased dramatically, and it almost completely disappeared from archaeological remains [26,27,28].

The major aims of this study were to identify the presence of Egyptian blue in the painting, map its distribution, and thus detect evidences of painted areas which were not visible or known before.

## Materials and methods

This study was conducted in Shivta National Park under the license of the Israel Antiquities Authority (G-87/2015, G-4/2016) and permit of Israel Nature and Parks Authority (6002/16).

### Visible Induced Luminescence imaging

VIL is a technique used to distinguish Egyptian blue from other natural or synthetic pigments. It is based on the fact that Egyptian blue luminesces strongly in the near IR spectrum at around 910 nm when illuminated by visible light [15,16].

The technique was used in order to detect and map Egyptian blue over the entire surface of the painting and to disclose missing details and hidden painted areas. As it is a non-invasive technique, it enabled us to obtain maximum data without touching the painted surface.

The VIL technique was implemented in an outdoor setting at night under complete darkness. It was easy to obtain these conditions, as Shivta is located in a remote desert area, far from any human settlements.

We calibrated the exposure and aperture settings of the camera for the VIL imaging, using three different types of standards, to select an optimal setting in all distances of photography between 0.5–4.0 m. These settings were based on a series of tests with different apertures and exposure times in order to maximize the ability to reveal details with the VIL imaging (see the photographs with standards at four different settings in S1 Fig).

We allowed on purpose minor stray IR radiation to be captured by the VIL imaging (S1C and S1D Fig) as a beneficial aid for determining the position of Egyptian blue with respect to the background niche and other features of the painting as proposed by Verri [17],

The following equipment was used:

■ Nikon D5100 DSLR camera modified by removing the inbuilt UV/IR-blocking filter equipped with an 18–55 mm f-3.5–5.6 Nikkor lens.
■ IR filter—Zykkor 52mm 850 nm high pass filter.
■ Excitation source—2 visible light LED sources were used to illuminate the painting surface, each with power output of 10W and luminance angle of 55°. The LEDs excitation wavelength is between 400–800 nm.
■ Calibration standards—these were used as a reference and control, mainly to check the presence of stray IR radiation in the images. Three types of standards were used: Labsphere Permaflect II^®^, La^b^sphere Spectralon^®^ and Datacolor Spyderchecker 24 (5%-99% reflectance standard) (S1 Fig).
■ Ocean Optics USB2000+XR Spectrophotometer was used to measure the wavelength of the light sources and the IR filter.
■ Nikon D7000 DSLR camera with a 60mm f-2.8 Micro Nikkor lens was used for comparative photographs of the areas examined.

### Instrumental analyses

Analyses of the pigments were carried out as part of an on-going study on the painting's materials and techniques. Egyptian blue was analyzed using several analytical techniques:

■ Optical Microscopy—A Leica DMRX Polarized Light Microscope was used for the optical analysis of samples. Cross-sections were prepared from samples (about 1x1 mm in size) mounted in polyester resin. The cross-sections were examined in incident light to analyze the paint layers, the color mixtures and the pigment particles.
■ SEM-EDS—a Zeiss Sigma HD scanning electron microscope with Oxford Instruments AZtec integrated EDS, X-Max 80, was used for high magnification imaging of the Egyptian blue pigment particles and for elemental analysis of their content.
■ Micro-Raman Spectroscopy—a custom-built portable instrument with 785 nm excitation, 50x objective and Si-CCD cooled detector was used. Typical acquisition was carried out with a 1200 grooves/mm grating with a power of less than 200–1500 Wcm^-2^ on the surface of the sample. Raman spectra were recorded in the spectral region from 150 to 3000 cm^-1^ with a spectral resolution of 10 cm^-1^. Raman measurements were carried out with an acquisition time of 5–20 s. The identification of Egyptian blue samples from Shivta, was made by comparison with Raman data from published databases and literature [29].

## Results and discussion

### Identification of Egyptian blue

The Egyptian blue pigment was identified using several techniques: optical microscopy, SEM-EDS, Raman spectroscopy and VIL.

**Fig 4:**
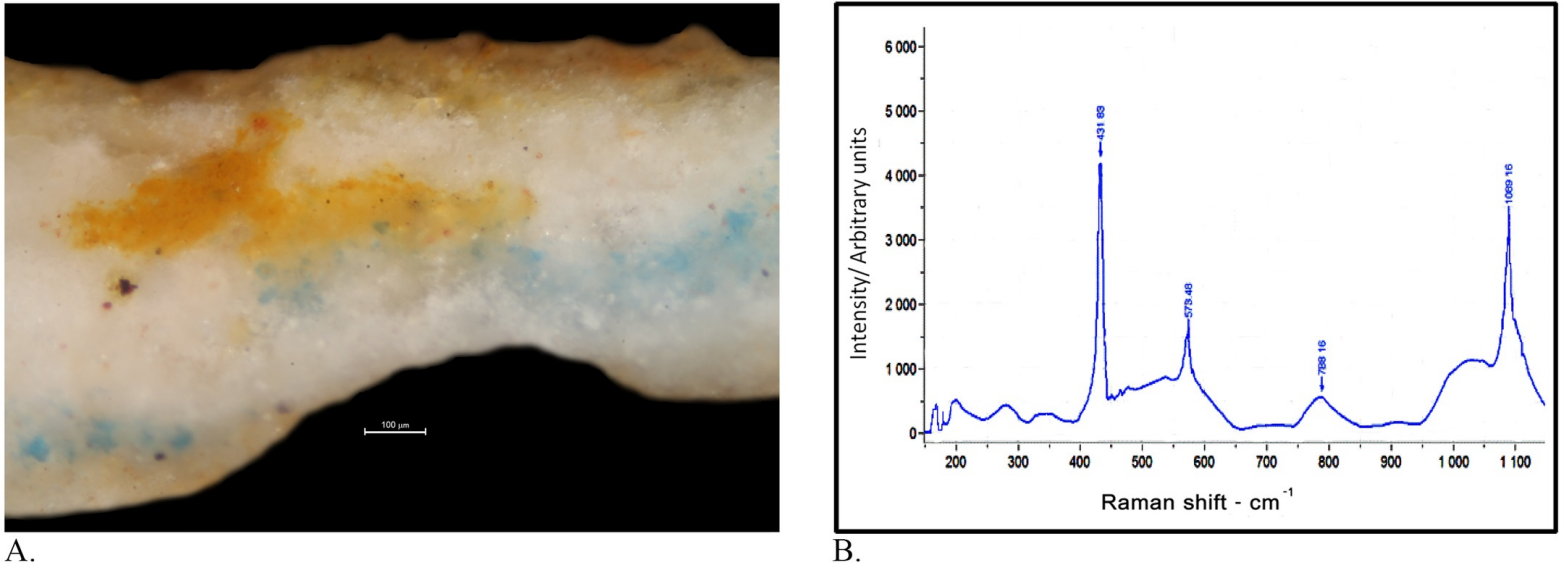
Instrumental analysis results. a: Micrograph of a cross-section of sample Sh-3 that was taken from the back of John, showing the Egyptian blue particles as a lower paint layer below two other paint layers–yellow in the middle and pink as the upper paint layer. This area looks pink to the observer (see Fig 3D) (Photo: R. Linn, 2016). b: Raman spectrum of Egyptian blue with peaks at 431, 573, 788 and 1089 cm^-1^.

The pigment particle size was examined by optical microscopy. The measured size of the Egyptian blue particles in the samples is between 20–40 μm (Fig 4A).

The SEM-EDS results confirmed the identification of Egyptian blue by elemental analysis that revealed the presence of Ca, Si, Na and Cu. It also confirmed the dimensions and structural characteristics of the Egyptian blue particles.

Micro-Raman spectroscopy analysis clearly demonstrated the presence of Egyptian blue, showing distinctive peaks at 431, 573, 788 and 1089 cm^-1^, typical Raman spectra of Egyptian blue (Fig 4B) [29]. In addition, fluorescence spectrum centered at 1500 cm^-1^ (890 nm) was acquired with the Raman instrument, identifying Egyptian blue from its strongest emission of Cu^2+^ [15,16,17].

**Fig 5:**
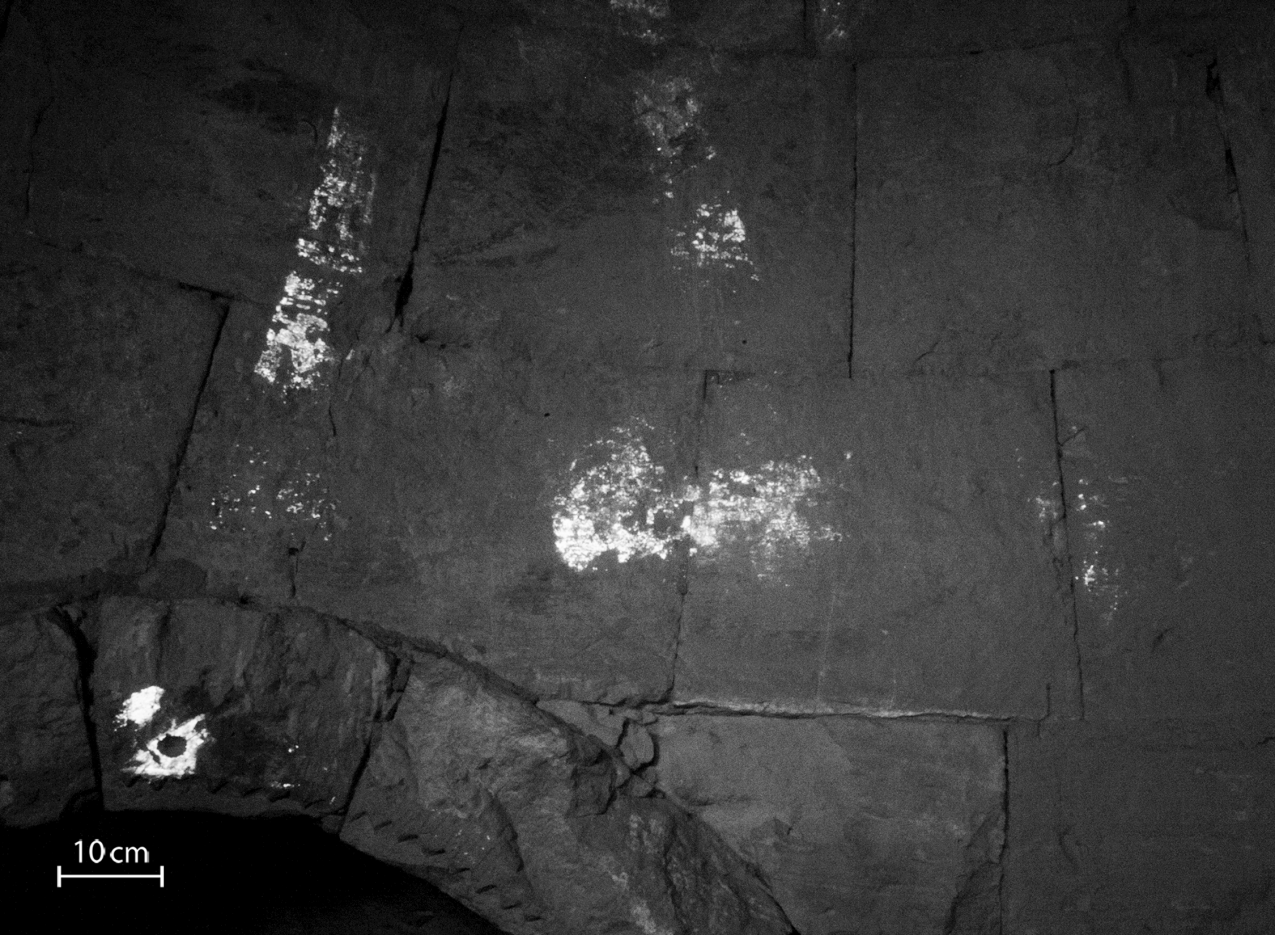
VIL image of the lower right part of the Transfiguration wall painting. The image shows the intense emission of areas painted with Egyptian blue, which appear in very bright white (Photo: R. Linn, 2016).

The VIL showed positive identification of areas that were painted with Egyptian blue, which were clearly observed from its very intense emission in the near IR spectrum that appeared as very bright areas in the VIL images [15,16] (Fig 5).

The identification of Egyptian blue is significant for the understanding of the use of the pigment during the Byzantine period. Although the pigment was extensively employed during the Roman period between the 1st century BCE and the early 4th century CE [23,24,30,31,32], until recently it was assumed that it had disappeared completely in the early Byzantine period. This conception has been re-evaluated in view of a very few findings of Egyptian blue in post-Roman wall paintings of the 7th to the 9th centuries CE [33,34], including St. Clemente in Rome [27,34,35]; St. Maria in Castelseprio (Lombardy) [36,37]; St. Maria Antiqua in Rome [34]; and Müstair in Switzerland [38,39]. These studies proved on one hand the existence of the pigment, but on the other hand confirmed its rarity during the Byzantine period [34].

The findings of this study show that Egyptian blue was still in use in the eastern Mediterranean after the Roman period, as reflected in the extensive presence of the pigment in large areas in the painting. Therefore, the identification of Egyptian blue in the Transfiguration painting at Shivta adds an important landmark that fills a gap in the historical chronology of the pigment's use during the first centuries after the Roman period.

### Distribution mapping of Egyptian blue in the painting

As described above, VIL imaging was used to map the distribution of Egyptian blue in the entire painting, in order to identify areas and patterns that were not otherwise observable. The technique enabled the detection of Egyptian blue *in-situ* in areas where no paint could be detected by the naked eye (Fig 5), as well as areas with paint that did not appear blue to the naked eye (Figs 3D and 4A).

It is important to emphasize that Egyptian blue was detected throughout the painting as a lower paint layer that reveals a clear pattern. It was used in that way, either as a sketch for the painting, or in order to attain the desired tones of color (Fig 4A).

#### Rays of light

**Fig 6:**
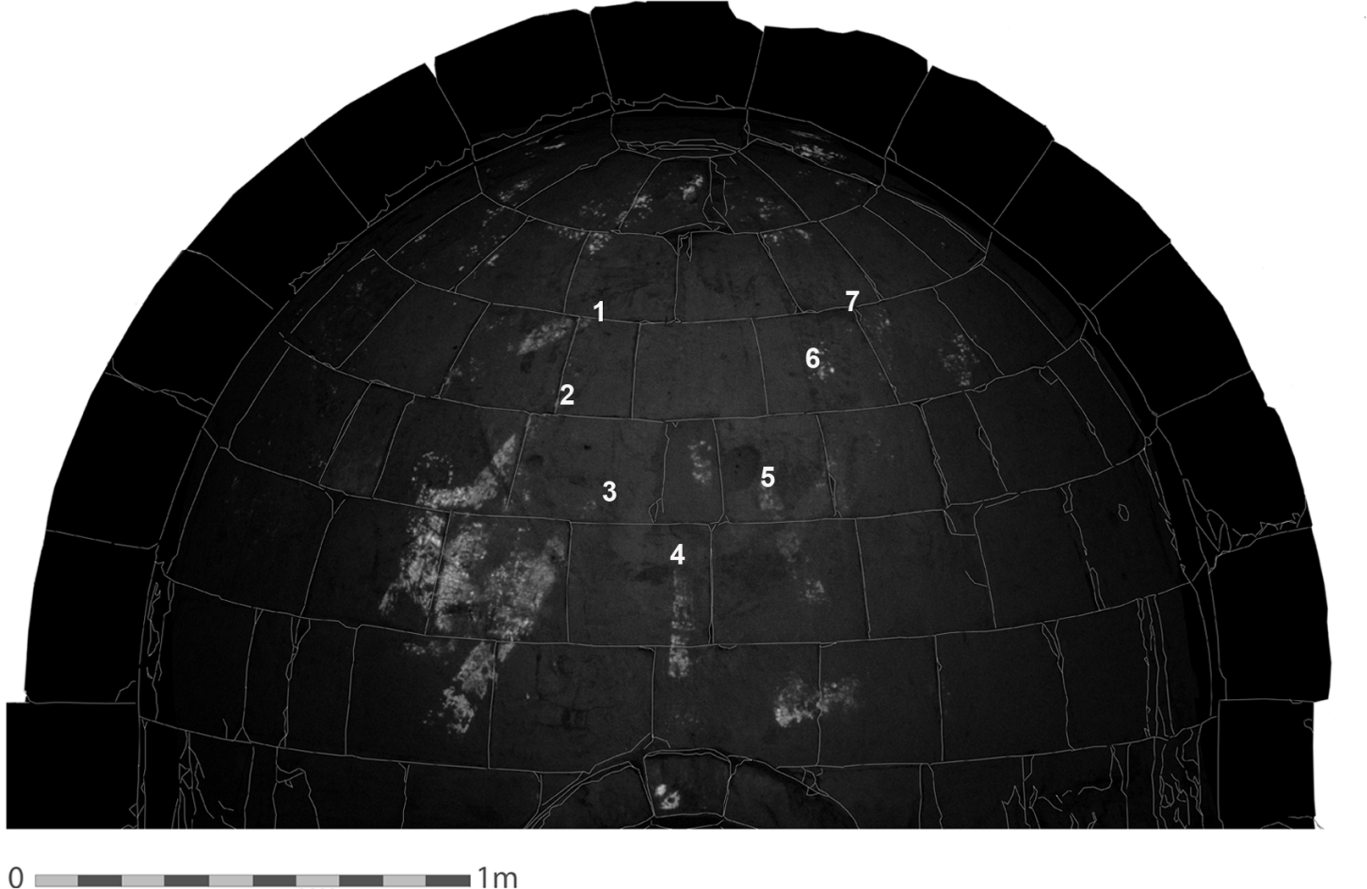
VIL image of the southern apse and the painted area. The VIL imaging of the upper part of the apse reveals very substantial details that are invisible to the naked eye and have never been detected before. The numbers indicate the rays of light. The stone blocks contour was added in order to give a better orientation where the rays and figures are located in the apse (Photo: R. Linn, 2016).

The most surprising and intriguing element detected by the VIL imaging is the iconographic motif of the rays of light that emerge from Christ to the other figures in the Transfiguration scene numbered anti-clockwise (Fig 6, Table 1).

**Table 1:** Description of the rays of light.

Ray 1	At an angle of 245°.Points down towards the figure to the left of Christ (either Elijah or Moses). Due to the poor preservation of the painting, this ray is clearly visible only from about half-way from the figure of Christ.
Ray 2	At an angle of 215°.Emerges from Christ, enters the figure of Peter below the head and continues through the body and chest down towards the right hand.
Ray 3	At an angle of 205°.Entering the figure of John in the area of the back and passes through the body down to the legs.
Ray 4	At an angle of 180°.Emerges from Christ towards the central stone of the niche.
Ray 5	At an angle of 160°.On the right of the figure of Christ, probably connects to the missing figure of James.
Ray 6	At an angle of 140°.Traces of a possible ray that is hardly visible were detected on the right of Christ.
Ray 7	At an angle of 115°.Traces of a possible ray on the right side of Christ were discovered opposite to the upper ray on his left side, at about the same level. The traces of this possible ray point towards another missing figure (either Moses or Elijah) on the right of Christ

As five rays (Fig 6, rays 1–5) are clearly visible with the VIL technique and traces of two possible additional rays (Fig 6, rays 6 and 7) were also detected in the painting, it is likely that the total number of rays that emerge from Christ was seven, or maybe even eight, corresponding to the 6th Century CE mosaic of the Transfiguration scene at the Monastery of St. Catherine [6,40] (Fig 1).

It is important to note that all the rays detected in the transfiguration painting at Shivta are pointed downwards towards the figures of Elijah, Moses, and the three disciples, and none of them is directed upwards (Fig 6). This contrasts the St. Catherine mosaic (Fig 1) and other Transfiguration scenes, where the rays towards Elijah and Moses point upwards.

It should be emphasized that there is a significant color difference between the background of the upper apse, above the two upper light rays (Ray 1 and Ray 7), which is considerably paler compared to the darker lower part of the apse (Fig 3). The difference may be related to the artists’ intention to separate between the upper sphere were the upper body of Christ, Elijah and Moses are depicted, and the lower area in which the three disciples are painted. Such separation between the upper and lower areas of the Transfiguration is also shown in the St. Catherine and St. Apollinare mosaics.

The light rays are a prominent motif of the Transfiguration scene in the St. Catherine mosaic and other later depictions of the Transfiguration [7,40]. Former descriptions of the Shivta scene lack any evidence of light rays emerging from the body of Christ. Moreover, Figueras [7] emphasized the absence of rays in this painting: "It is interesting that in Shivta no light rays seem to have been painted as coming from the glorious body of Christ, as in Sinai and everywhere else". The discovery of the rays in this study fills a gap in our knowledge and understanding of the Shivta painting.

**Fig 7:**
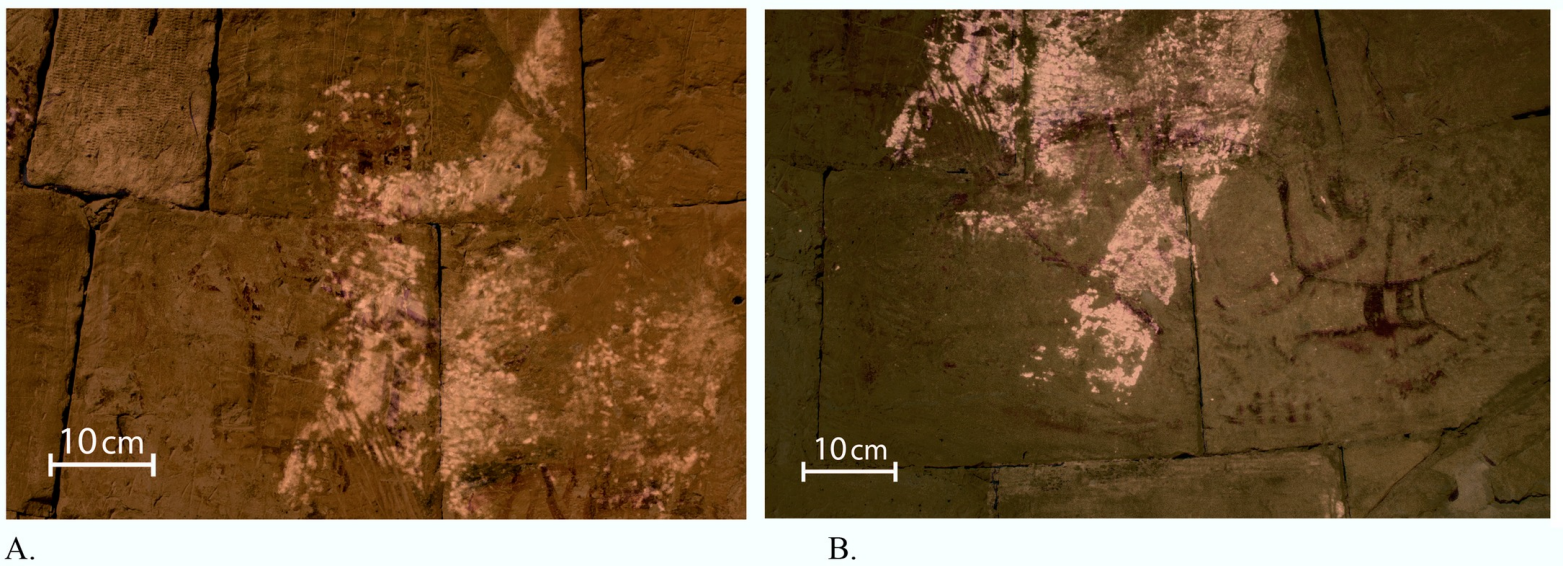
**Superimposed VIL images and regular photographs of Peter (a) and John (b).** Significant elements which were revealed for the first time by the VIL imaging are combined together with the lines and colors of the regular photography, showing their actual location in each figure. Note the use of Egyptian blue to depict Peter's hair and the complete light ray that enters John's body (Photo: R. Linn, 2016).

The use of Egyptian blue for depicting the rays of light, although it was painted underneath other colors and not visible to the naked eye (Fig 3B, 3C and 3D, Fig 4A, Fig 6, Fig 7), might have a religious connotation. This corresponds with the use of the color blue in the apse at St. Catherine’s monastery in Sinai, where the tunics of Peter and James turn deep blue where Christ’s rays strike them [41] (Fig 1).

Thus, this special use of Egyptian blue in the Shivta Transfiguration wall painting might have a religious meaning and symbolism.

When discussing the meaning of the color blue in Byzantine art, Gage [41] suggests that: "… in the Gospel accounts of the Transfiguration, blue is the color of the Divine Darkness which transcends light”. This remark highlights the intentional use of the color blue in the Transfiguration scene as representing transcendent light, and this is possibly one of the reasons why it was used by the artists in Shivta to depict the rays that emerge from Christ.

The Gospel of John differs from the Synoptic account of the Transfiguration by identifying Jesus as “the light” (vv. 4f, 7), and by announcing that “In him was life, and the life was the light of all people” (v. 4) [42]. The distinctive rays of light emanating from the figure of Christ that lit each of the five witnesses to the vision of the Transfiguration are a key element in this scene [40].

The Transfiguration scene involves as a central element a bright, divine light gleaming from Christ‘s face and clothes, a light that orthodox Christianity has referred to as the “uncreated light” [43]. The Transfiguration scene of the mosaics at the Monastery of St. Catherine in Sinai is the earliest known example of this scene with rays of light. Neither the rays nor the mandorla appear in two earlier representations of the Transfiguration: the Brescia casket and the wooden doors of Santa Sabina in Rome [40]. The revelation of the light rays in the Shivta Transfiguration wall painting, like the scene in the St. Catherine monastery mosaics, highlight the importance of the motif of the rays and its use in the Transfiguration scenes of that early Christian period.

#### The mandorla

**Fig 8:**
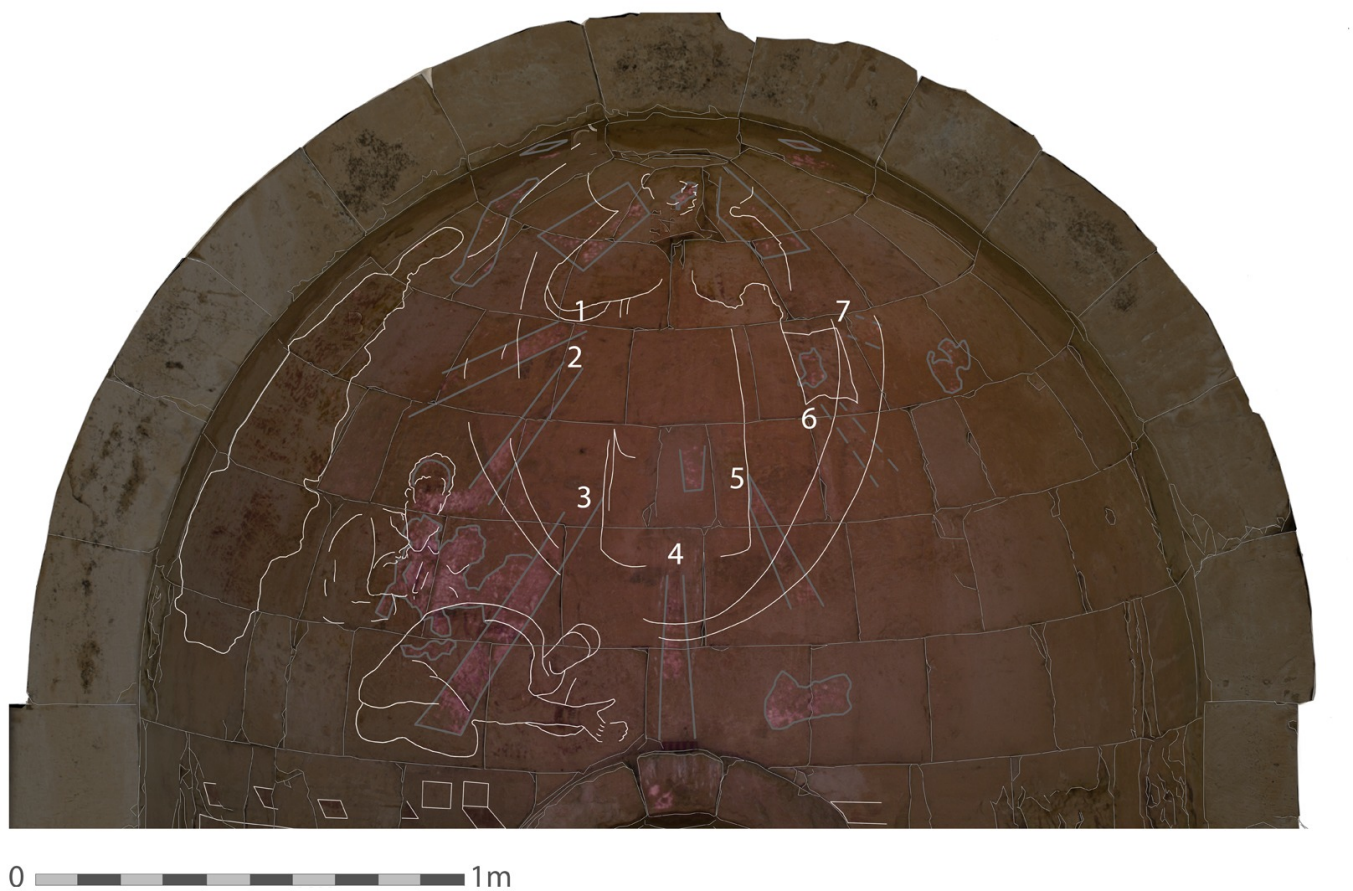
Superimposed image of the southern apse. This image shows a photograph of the entire painting combined with the VIL image with emphasized digital reconstruction of the outlines of the figures and the motifs. This combined image shows for the first time the invisible and visible details together and reveals the original composition of the scene (Photo: R. Linn, 2016).

Another key element in the Transfiguration scene is the mandorla surrounding the figure of Christ, which is not well preserved and hard to define in the Shivta painting. A blue color is not visible inside the mandorla, but through VIL imaging, remains of Egyptian blue were discovered in different parts in the outer strip of the mandorla. It is likely that the outer strip of the mandorla was based on Egyptian blue. In addition, the VIL images enabled us to note the two thin lines of a dark color that were painted above the Egyptian blue and define the width of the outer mandorla strip (about 12 cm). This phenomenon appeared, in areas where the rays cross the mandorla outer border, e.g. in ray 2, where the two dark lines are emphasized by the bright background of the Egyptian blue emission (Figs 6 and 8).

The use of blue color for depicting elements of the mandorla around Christ is documented in various other works of art of the Transfiguration scene, including the mosaics of St. Catherine, Ravenna and other later Byzantine works of art: in the Carolingian wall painting of Müstair in Switzerland, of 800 CE [44] and in the painting of the Ascension of 847–850 CE in the St. Clemente church in Rome [27,35].

#### The figures

The use of VIL imaging was highly important in revealing Egyptian blue in the depiction of the different figures of the scene as shown in Figs 7 and 8 and Table 2.

**Table 2:** Location of Egyptian blue in the figures of the Transfiguration at Shivta and its significance.

**Christ**	Egyptian blue was found in the face and the upper body of Christ. In addition, from the two sides of Christ’s head, appear two triangular areas depicted with Egyptian blue (S3 Fig). The use of Egyptian blue in the center of Christ’s face probably enabled the artists to obtain the right tone color to reflect the enlightenment of Christ. This finding corresponds to other descriptions of the use of Egyptian blue, such as in the painting of St. Maria in Castelseprio, Lombardy, where it was interpreted as under-painting for flesh tones [34]. The triangular areas that appear from the two sides of Christ’s head, probably form part of the upper garment that covered Christ’s shoulders. This detail of the Shivta painting has been described by Woolley and Lawrence in 1914 [14]: “The *chiton*…of light pink edged with gold, the *himation* of dark blue” (S1 Text).
**John**	The most prominent use of Egyptian blue is a very distinctive ray of light passing through the entire figure of John (Fig 6, ray 3), another area depicted with Egyptian blue was detected in the back of John (Fig 7B). A detailed observation suggests that the pigment was used in parallel with the folds of John’s garment, a use that was found in other Byzantine religious works of art [34]. The way the ray of light passing through John's body (Fig 7B) is similar to the way the rays of light penetrate the body of the figures in the Transfiguration scene of St. Catherine in Sinai (Fig 1).
**Peter**	A unique concentration of Egyptian blue was found in the area of the figure of Peter (Fig 7A): It appears in Peter’s beard and in the lower part of his face. A horizontal strip of Egyptian blue joins the ray coming from the upper right side from Christ’s body. This horizontal strip could be a breaking of the ray of light in order to illuminate Peter’s face (Fig 7A). In addition, Egyptian blue is also used in a fold of the right sleeve of his garment. The use of Egyptian blue for folds of garments is found also in the Carolingian wall painting of Müstair in Switzerland of 800 CE [34,44]. An interesting use of Egyptian blue is in the form of a rounded crown marking the area of the hair of Peter (Fig 7A). The Egyptian blue in the hair of Peter corresponds to an earlier similar use of it in the mummy portraits from Fayum and Tebtunis, Egypt, dated to the Roman period, in the 2nd century CE [17,20].
**James**	A very clear ray (Fig 6, ray 5) points towards the probable location of the figure of James that was presumably depicted at the lower right side of the scene. Clear remains of Egyptian blue were also detected in the probable location of James's figure (Fig 5). The painting on the southern side of the apse is totally destroyed and shows very little trace of color, thus due to the detection of Egyptian blue pigment it was possible to assume the figure location.
**Elijah and Moses**	Egyptian blue was detected as multiple small dots covering the whole figure to the left of Christ, under painted areas of yellow and red (S2 Fig). Although the figure is totally damaged, yet these traces of color revealed by the VIL imaging enable us to detect the location of the figure and to suggest the use of Egyptian blue as the lower paint layer (S2 Fig).

#### The design at the upper border of the apse

This area is covered with thick incrustation that does not allow the determination of a clear pattern of decoration, although some traces of color appear where the incrustation is missing. VIL imaging revealed part of the original decoration at the edge of the apse which appears as a geometric pattern of parallelograms depicted in Egyptian blue (S3 Fig).

#### The niche

There is a niche in the lower part of the center of the apse with an arch decorated with triangles in alternating colors. Only traces of colors can be seen today. These decorations were described by Woolley and Lawrence in 1914 [14]: "the tooth pattern round the arch of the small recess was picked out in red and blue…the recess in the northern apse was similarly decorated" [14].

VIL imaging shows Egyptian blue in one of the triangles, at the center of the arch of the niche (Fig 5, lower left corner). Similar decorations with Egyptian blue were discovered in the northern apse of the church as discussed below, where the pigment is clearly detected on six triangles on the arch of the niche (S4 Fig), corresponding well with the description of Woolley and Lawrence (S1 Text), and confirming that both niches were similarly decorated with red S4 Fig aand blue (S4B Fig).

### Distribution mapping survey of Egyptian blue in the other apses of the southern church at Shivta

An initial survey of the two other apses at Shivta’s southern church revealed very slight remains of wall paintings that, together with a literature survey, indicate that these apses were also painted.

In the northern apse, except the red triangle decoration, no clear painting or colors were visible. As mentioned above, the VIL imaging technique also detected clear remains of the decoration, corresponding well with the description of Woolley and Lawrence [14], and confirming that the niches in both apses were similarly decorated as described above.

Other parts of the northern apse also reveal patterns of painted areas of Egyptian blue, such as remains of possible rays. Traces of Egyptian blue throughout the entire surface of the apse indicate that it was probably painted in a similar technique and style to the southern apse.

On the contrary, in the central larger apse, only traces of a very small fragment of blue-painted plaster (about 5x5 cm) were found. This blue color was analyzed with VIL imaging, indicating that it was probably painted with a different blue pigment than Egyptian blue. This supports the fact that this area was painted on plaster, using a different technique than the southern apse of the Transfiguration scene.

### Examination of the painting technology

VIL Imaging was also used to study the application techniques of the Transfiguration painting: although the original distribution of the blue color was not visible, the restricted area in which it should have been painted was well planned ahead. The Egyptian blue layer that was painted below other colors (Fig 4A), created a clear pattern that could be detected only with VIL imaging. The phenomenon of layering of the colors was consistent in the entire painting, and was used to sketch the painting areas or to achieve a specific tone or for other reasons, such as an iconographic connotation as described above. Due to the fact that the painting remains are very fragmentary, in some areas the pattern of the upper paint layers were detected better on top of the luminescent areas of Egyptian blue which emphasized the lines and design; e.g. in John's back and in the Mandorla outline to the left of Christ (Fig 6, Fig 7). Such use of Egyptian blue as a lower paint layer or background of paintings is well documented in later Byzantine religious works of art, as discussed above [27,34,35,44].

### Conservation aspects detected by VIL imaging

VIL imaging provided important information on areas where the painting was damaged or no longer visible, such as in the southern half of the apse (Fig 5), where colors are missing; in the face of Christ (S3 Fig) and in paint layers that were covered by heavy incrustation, e.g. in the parallelograms at the upper area of the apse (S3 Fig). The fact that Egyptian blue layers were applied below other paint layers may be the reason that it was better preserved than other pigments. Due to its remarkably strong luminescence, it was possible to detect traces of Egyptian blue pigment particles even in areas where the painting is worn away and no other pigment was detected.

An important discovery by the VIL imaging is related to repairs of a stone block in the southern apse, indicated by the presence of modern cement. The VIL image of Ray 4, shows a clear break in the line of the ray, which might indicate that during former restoration works the stone was probably put back in the wrong orientation (S5A Fig). In our digital reconstruction of the stone's position, we rotated it by 180° and as a result, there is a clear continuation of the ray (S5B Fig).

## Conclusions

This study used VIL imaging to investigate the presence of Egyptian blue pigment in the early Christian wall painting of the Transfiguration scene in Shivta. It showed that the pigment was available to the artists of Shivta in the early Christian period. This technique proved to be a very efficient tool for identifying Egyptian blue and mapping its distribution on the painted surface as well as an under-drawing.

The results of the study clearly show that Egyptian blue was found in areas that were not previously identified as being painted by any other method, and also show the pattern of painted areas that were impossible to interpret before.

The most intriguing outcome of this study was the detection of the light rays that emerge from the body of Christ to the other figures in the scene, a result that has highly significant iconographic and theological importance.

The significance of the findings is mainly in the new and exciting information, which provides very important highlights to our understanding of the oldest known wall painting of the Transfiguration scene. VIL imaging is thus shown to be an efficient technique for detailed studies and surveys of wall paintings in archaeological sites where there was extensive use of Egyptian blue.

## Supporting information

S1 Text Citation from: Woolley, C.L. and Lawrence, T. E. 1914. "The Wilderness of Zin (Archaeological Report)". Palestine Exploration Fund DS111.A1P28, Vol. 3 copy 1. Harrison.
(PDF)

S1 Fig Four VIL photos with Labsphere Permaflect II^®^ calibration references. a: Exposure time– 1/10 s. f-number 8. No stray light–all references are black. b: Exposure time– 1/6 s. f-number 8. Very little stray light at the bottom of the photograph, references of 5% and 18% are exposed. c: Exposure time– 1/4 s. f-number 8. Stray light appears–references of 5% up to 50% are exposed. d: Exposure time– 1/2 s. f-number 8. More stray light is seen–references 5% up to 50% are exposed. (Photo: R. Linn, 2016).
(TIF)

S2 Fig VIL image of the dots of Egyptian blue that emerged in the area of the figure left of Christ below the red and yellow visible colors(Photo: R. Linn, 2016). (TIF)

S3 Fig VIL image from the surrounding of Christ's face, showing the probable *himation* area reconstructed with white outlines. The geometric pattern of parallelograms in the outer border of the apse is depicted with Egyptian blue and thus revealed only by the VIL technique through the thick incrustation (Photo: R. Linn, 2016).
(TIF)

S4 Fig VIL image of the triangles decorations in the northern apse. The arch of the inner niche of the northern apse showing that the downward pointing triangles are red in regular photography (a) and the upward pointing ones have traces of Egyptian blue shown only in the VIL image (b) (Photo: R. Linn, 2016).
(TIF)

S5 Fig Astone block that has traces of ray 4 revealed by VIL imaging, showing that it was formerly put in place wrongly (a). A digital reconstruction shows its correct position (b) (Photo: R. Linn, 2016).
(TIF)
