# Supplementary material for: Visible induced luminescence reveals invisible rays shining from Christ in the early Christian wall painting of the Transfiguration in Shivta
Source: PLoS One. 2017 Sep 26;12(9):e0185149. doi: 10.1371/journal.pone.0185149 (PMC5614614; doi:10.1371/journal.pone.0185149)
Supplement: S1 Text — "The Wilderness of Zin (Archaeological Report)". Palestine Exploration Fund DS111.A1P28, Vol. 3 copy 1. Harrison. (PDF) [file pone.0185149.s001.pdf]

Description of the painting at Shivta, by Woolley, L.C. and Lawrence, T. E.
The Wilderness of Zin (Archaeological Report), PEF, Harrison and Sons, London. 1914. (pp. 89-90).

"In the southern apse alone could any coherent design be distinguished, and here the colours had faded under exposure to the light, most of the surface had been scraped away by iconoclasts, and rain-water had brought down lime from the upper ruins and left a thick white deposit over the whole wall-face. Only by wetting the stone were we able to make out and roughly to sketch the original painting. The subject was the Transfiguration.

In the centre is Christ, full-face, with hands raised and brought together over the breast, The *chiton* was seemingly of light pink edged with gold, the *himation* of dark blue; the halo was a plain yellow ring with white centre; the *vesica* of light pink. The figure was too much damaged to be copied.

Below the feet is a semi-prostrate figure, probably of St. Peter, and beyond, on the spectator's left, a kneeling figure identified by a fragmentary inscription …ANNIC, in red paint, as S. John; turning half-round to the front, he raises his left hand, as if pointing to Christ.

A few lines on the right of the vesical are all that is left of S. James. On either side of the apse, a little distance from the central group, a blurred mass of red colours seems to represent figures standing on a slightly higher level than the Apostles: these are probably Moses and Elijah.

Below the feet of the figures is a broad red band.

The tooth pattern around the arch of small recess was picked out in red and blue, and its vault was roughly painted in red with a coarse network pattern, each mesh having a cross as filling ornament."
