# Supplementary figures and images for: Visible induced luminescence reveals invisible rays shining from Christ in the early Christian wall painting of the Transfiguration in Shivta
Source: PLoS One. 2017 Sep 26;12(9):e0185149. doi: 10.1371/journal.pone.0185149 (PMC5614614; doi:10.1371/journal.pone.0185149)

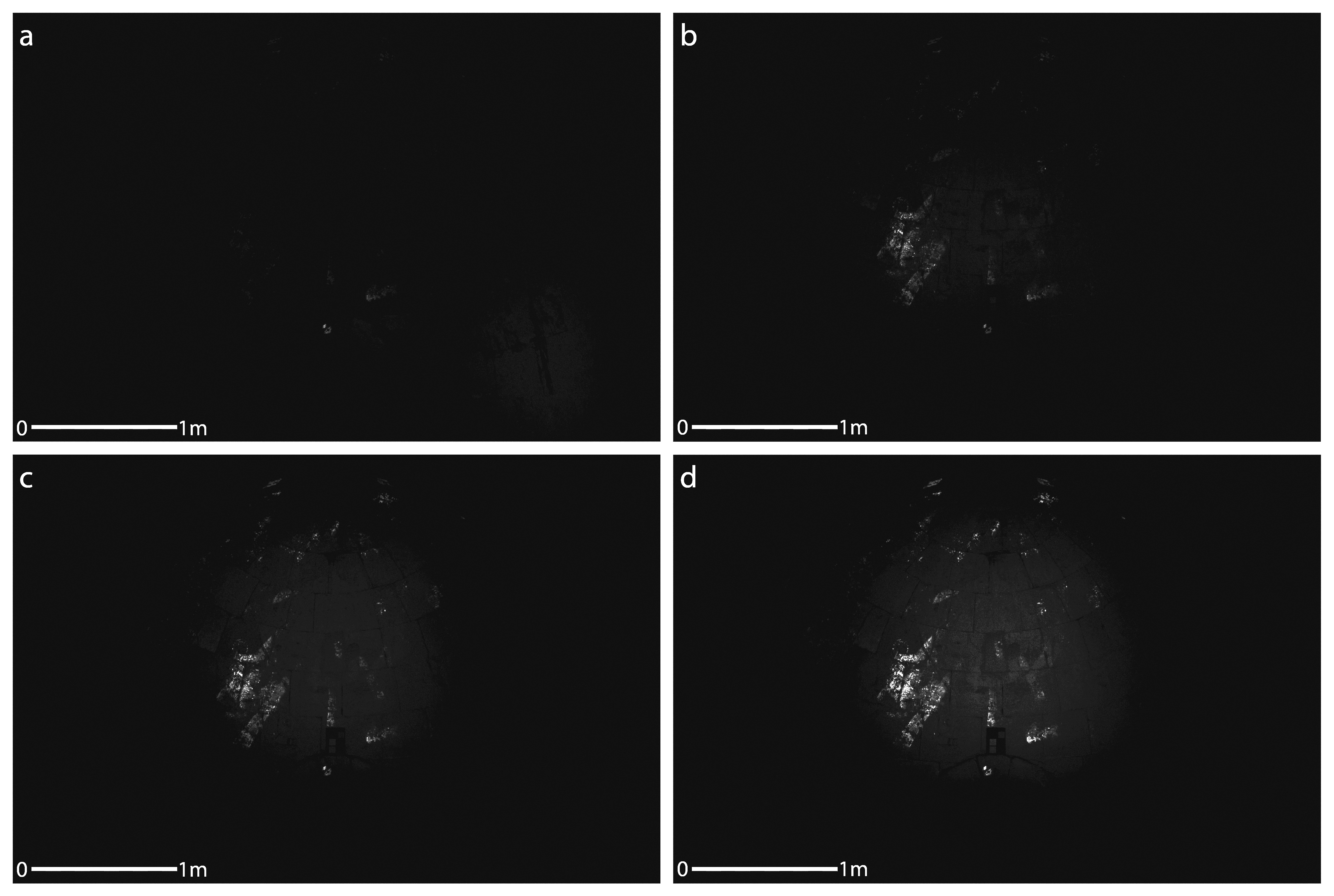

Supplement: S1 Fig — a: Exposure time– 1/10 s. f-number 8. No stray light–all references are black. b: Exposure time– 1/6 s. f-number 8. Very little stray light at the bottom of the photograph, references of 5% and 18% are exposed. c: Exposure time– 1/4 s. f-number 8. Stray light appears–references of 5% up to 50% are exposed. d: Exposure time– 1/2 s. f-number 8. More stray light is seen–references 5% up to 50% are exposed. (Photo: R. Linn, 2016). (TIF) [file pone.0185149.s002.tif]

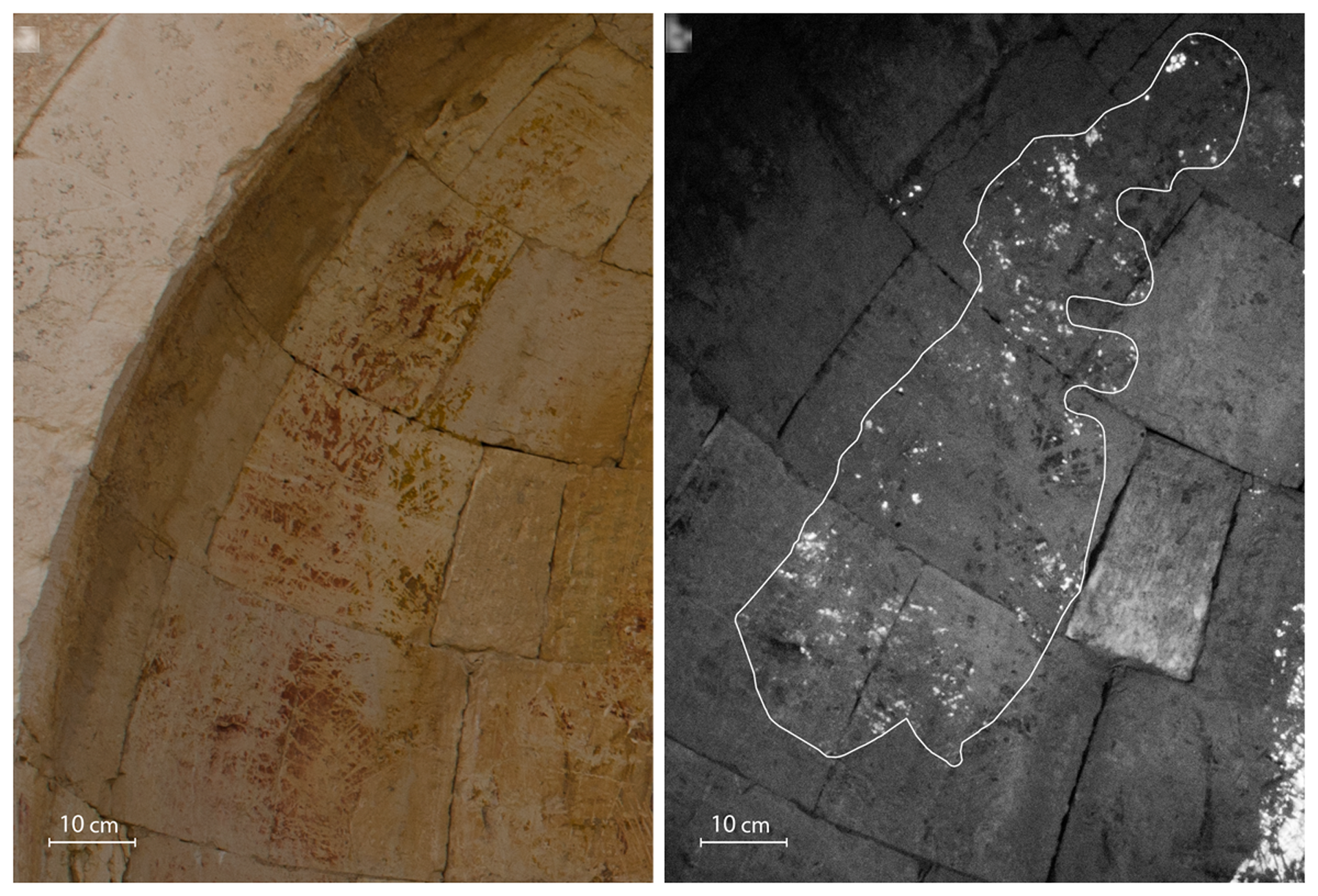

Supplement: S2 Fig — (TIF) [file pone.0185149.s003.tif]

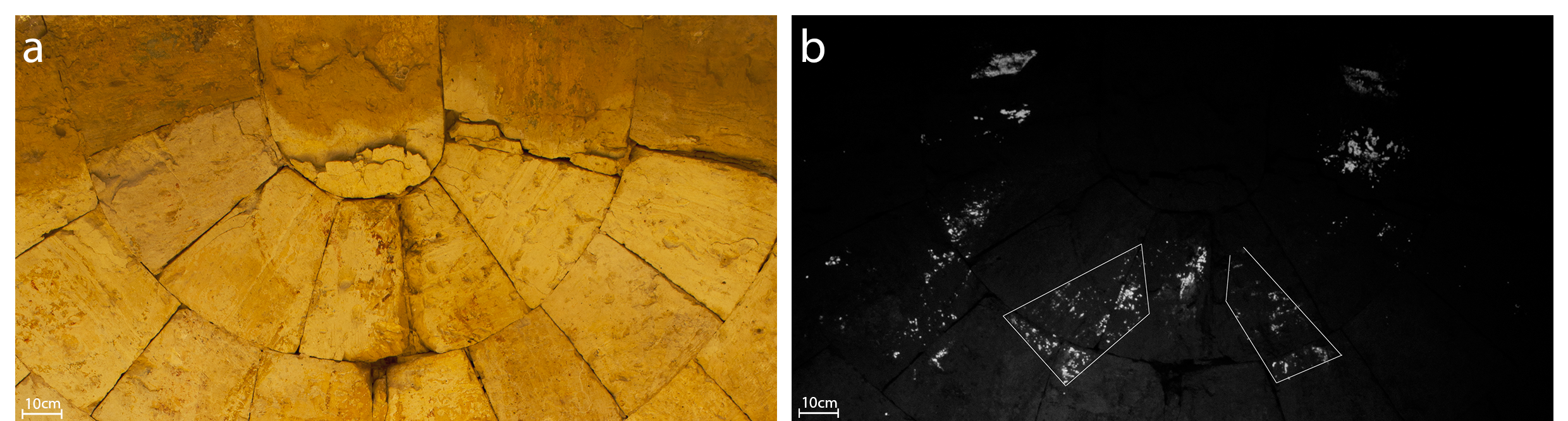

Supplement: S3 Fig — The geometric pattern of parallelograms in the outer border of the apse is depicted with Egyptian blue and thus revealed only by the VIL technique through the thick incrustation (Photo: R. Linn, 2016). (TIF) [file pone.0185149.s004.tif]

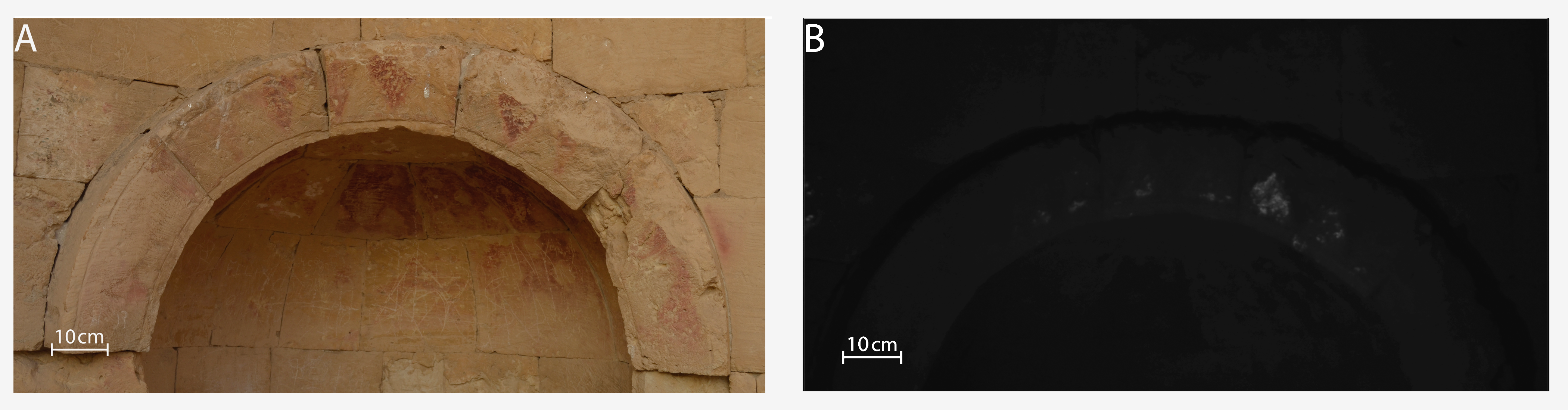

Supplement: S4 Fig — The arch of the inner niche of the northern apse showing that the downward pointing triangles are red in regular photography (a) and the upward pointing ones have traces of Egyptian blue shown only in the VIL image (b) (Photo: R. Linn, 2016). (TIF) [file pone.0185149.s005.tif]

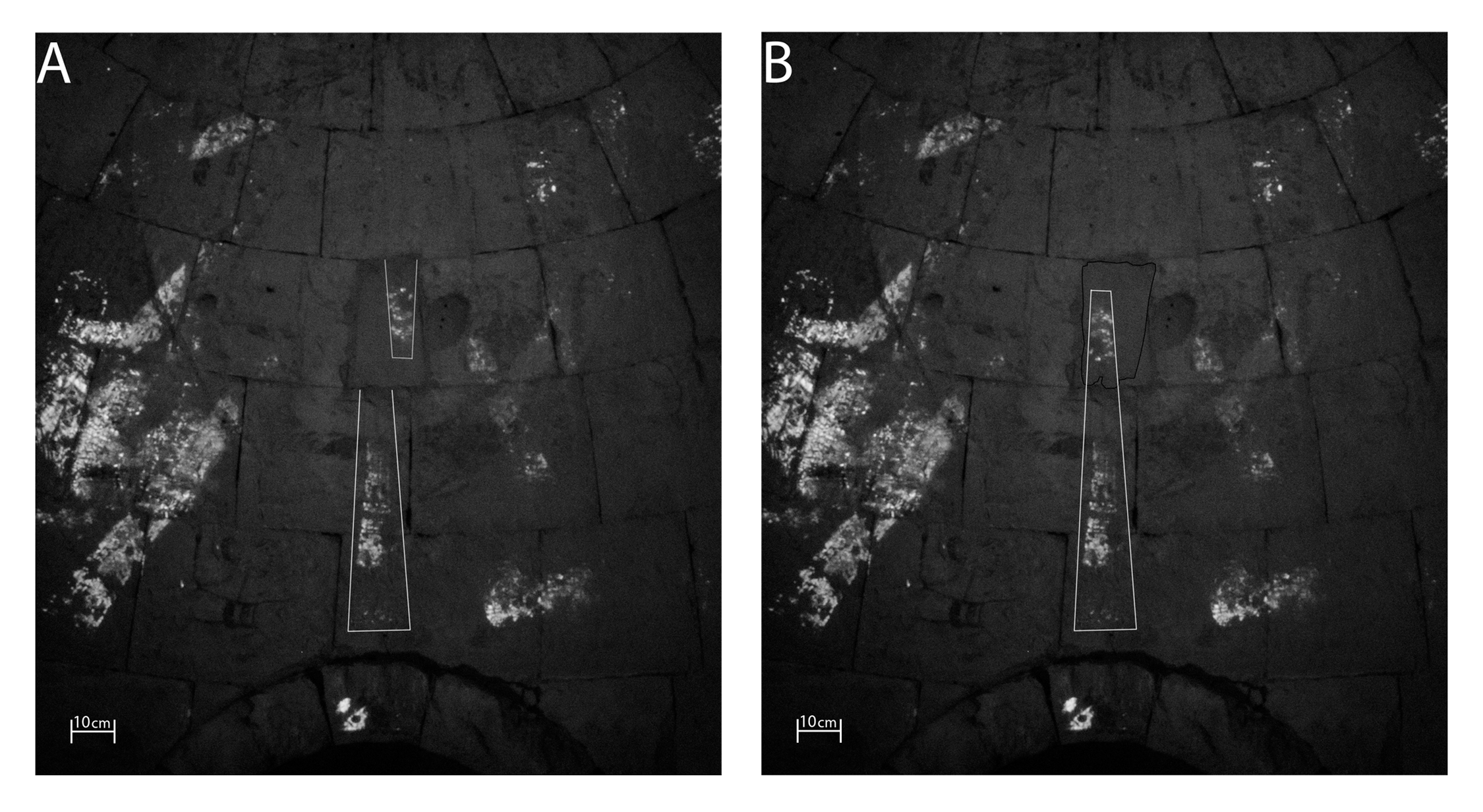

Supplement: S5 Fig — Astone block that has traces of ray 4 revealed by VIL imaging, showing that it was formerly put in place wrongly (a). A digital reconstruction shows its correct position (b) (Photo: R. Linn, 2016). (TIF) [file pone.0185149.s006.tif]
